# Deep learning for segmentation of the cervical cancer gross tumor volume on magnetic resonance imaging for brachytherapy

**DOI:** 10.1186/s13014-023-02283-8

**Published:** 2023-05-29

**Authors:** Roque Rodríguez Outeiral, Patrick J. González, Eva E. Schaake, Uulke A. van der Heide, Rita Simões

**Affiliations:** Department of Radiation Oncology, The Netherlands Cancer Institute, Plesmanlaan 121, 1066 Amsterdam, CX The Netherlands

**Keywords:** Automatic segmentation, Cervical cancer, GTV, Nnu-net, Brachytherapy

## Abstract

**Background:**

Segmentation of the Gross Tumor Volume (GTV) is a crucial step in the brachytherapy (BT) treatment planning workflow. Currently, radiation oncologists segment the GTV manually, which is time-consuming. The time pressure is particularly critical for BT because during the segmentation process the patient waits immobilized in bed with the applicator in place. Automatic segmentation algorithms can potentially reduce both the clinical workload and the patient burden. Although deep learning based automatic segmentation algorithms have been extensively developed for organs at risk, automatic segmentation of the targets is less common. The aim of this study was to automatically segment the cervical cancer GTV on BT MRI images using a state-of-the-art automatic segmentation framework and assess its performance.

**Methods:**

A cohort of 195 cervical cancer patients treated between August 2012 and December 2021 was retrospectively collected. A total of 524 separate BT fractions were included and the axial T2-weighted (T2w) MRI sequence was used for this project. The 3D nnU-Net was used as the automatic segmentation framework. The automatic segmentations were compared with the manual segmentations used for clinical practice with Sørensen–Dice coefficient (Dice), 95th Hausdorff distance (95th HD) and mean surface distance (MSD). The dosimetric impact was defined as the difference in D98 (ΔD98) and D90 (ΔD90) between the manual segmentations and the automatic segmentations, evaluated using the clinical dose distribution. The performance of the network was also compared separately depending on FIGO stage and on GTV volume.

**Results:**

The network achieved a median Dice of 0.73 (interquartile range (IQR) = 0.50–0.80), median 95th HD of 6.8 mm (IQR = 4.2–12.5 mm) and median MSD of 1.4 mm (IQR = 0.90–2.8 mm). The median ΔD90 and ΔD98 were 0.18 Gy (IQR = -1.38–1.19 Gy) and 0.20 Gy (IQR =-1.10–0.95 Gy) respectively. No significant differences in geometric or dosimetric performance were observed between tumors with different FIGO stages, however significantly improved Dice and dosimetric performance was found for larger tumors.

**Conclusions:**

The nnU-Net framework achieved state-of-the-art performance in the segmentation of the cervical cancer GTV on BT MRI images. Reasonable median performance was achieved geometrically and dosimetrically but with high variability among patients.

**Supplementary Information:**

The online version contains supplementary material available at 10.1186/s13014-023-02283-8.

## Background

For locally advanced cervical cancer the standard of care consists of external beam radiotherapy (EBRT), followed by 3 to 4 fractions of brachytherapy (BT) and concomitant chemotherapy [1]. A key step in both EBRT and BT treatment planning is the segmentation of organs at risk and target volumes. This is mostly performed manually, which is time consuming and suffers from the inherent bias of the observer. To circumvent these issues, automatic segmentation is being widely investigated in the field of radiotherapy [2–4]For the case of BT, the need for automatic segmentation is even more critical due to the time constraints of the workflow. At each fraction of BT treatment, the applicator is inserted surgically in the patient, after which the MRI images are acquired. The patient then needs to wait, immobilized in bed, while the needed structures (namely organs at risk and target volumes) are manually delineated and a treatment plan is made. The Gynecological (GYN) GEC-ESTRO working group defines the target volumes of interest for BT treatment planning for this cervical cancer as the Gross Tumor Volume (GTV), the high risk Clinical Target Volume (HR-CTV) and the intermediate risk Clinical Target Volume (IR-CTV) [5] and they are currently segmented by radiation oncologists. Automatic image segmentation methods are expected to reduce the clinical workload as well as patient burden.

Automatic segmentation of the targets volumes is still uncommon and it is mostly limited to positron emission tomography (PET) and/or computed tomography (CT) and rarely to magnetic resonance imaging (MRI) [9]. For the particular case of cervical cancer on BT images, automatic segmentation of the organs at risk has been widely investigated [6–10] but literature on the automatic segmentation of the targets, and especially the GTV, is more scarce [7, 9, 10]. Zhang et al. [10] and Wong et al. [9] developed automatic segmentation tools that segmented the HR-CTV (on CT and MRI images, respectively) but to the best of our knowledge, only Yoganathan et al. [7] have studied the automatic segmentation of the gross tumor volume (GTV) on BT MRI images. While they demonstrated that automatic segmentation of the GTV is possible in principle, the cohort was rather small with only 39 patients, resulting in a relatively weak performance with Sørensen–Dice coefficients (Dice) between 0.57 and 0.62. Furthermore, the segmentation architectures used in their project were based on the ResNet50 architecture[11], which is no longer considered state of the art.

A current state-of-the-art framework for the automatic segmentation of medical structures is the nnU-Net (‘no-new-U-Net’) [12]. The nnU-Net is a deep learning-based framework which automatically configures the parameters needed for training. It has been shown to outperform other approaches on 23 public datasets used on segmentation competitions.

The aim of this study was to assess the quality of the automatic segmentations of the cervical cancer GTV on BT MRI images. We used two methods to determine to what extent the automatic segmentations corresponded to the clinical segmentations performed by an expert radiation oncologist. First, the geometrical correspondence of the automatic and expert delineation was determined using Dice Similarity Coefficient (Dice), 95th Hausdorff Distance (95th HD) and mean surface distance (MSD). Then, to find if the observed geometrical differences between the delineations would have dosimetric consequences, we determined dose-volume parameters D90 and D98 for the automatic and expert delineations using the clinical dose distribution.

## Methods

### Data

A cohort of 195 histologically proven cervical cancer patients treated in our institution between August 2012 and December 2021 was retrospectively collected. The average age was 53 (standard deviation of 15 years) and tumor stage ranged from IB to IV according to the International Federation of Gynecology and Obstetrics (FIGO) staging [13]. The treatment consisted of external beam radiotherapy (156 patients with 23 × 2 Gy and 39 patients with 25 × 1.8 Gy) followed by BT (3 × 7 Gy) and combined with chemotherapy (cisplatin 40 mg/m2, weekly). The institutional review board approved the study (IRBd20276). Informed consent was waived considering the retrospective design.

A total of 524 separate BT fractions were included in this work. For each BT fraction, MRI images of the patient with applicator in place were acquired using a 1.5T (104 scans) or 3T (442 scans) Philips Medical Systems MRI scanner. Axial T2-weighted (T2w) turbo spin-echo images were used (TR =[3500–13,300 ms], TE = [100–120 ms]) with a pixel spacing of 0.39 mm x 0.39 mm (442 scans) or 0.63 mm x 0.63 mm (104 scans) and a slice thickness of 3 mm. The GTV, as segmented for treatment planning by a radiation oncologist on each available MRI, was available as ground truth.

The data set was split into three subsets at the patient level: training set (117 patients, 314 images), validation set (39 patients, 104 images) and test set (39 patients, 106 images). The three subsets were stratified according to FIGO stage [13], because it is a relevant clinical parameter used to describe gynecological tumors.

### Network architecture and training procedure

The nnU-Net framework was used in this work. This framework automatically configures the parameters needed for preprocessing, network architecture and training for each specific task. The loss function was a combination of the Dice loss [14] and cross entropy loss. We used the stochastic gradient descent (SGD) optimizer with learning rate scheduler and early stopping based on the validation loss as criterion to choose the best model. Dropout, data augmentation and weight decay were used as regularization techniques. Further details on the training procedure can be found in the Additional file 1.

### Experiment overview

#### Geometric comparison

The automatic segmentations were compared to the manual segmentations of the GTV that were performed by a radiation oncologist for treatment planning for the patients on the separate test set. The automatic segmentations were compared to the manual segmentations using common segmentation metrics: Dice, 95th HD and MSD, which were implemented using the Python package by DeepMind (https://github.com/deepmind/surface-distance). The segmentation results were additionally compared among patients with different FIGO stage and GTV volume. For the volume analysis, the patients of the test set were allocated to four volume ranges containing the same number of images in each bin.

Attention maps were computed for four different examples to highlight which parts of the input image were relevant for the network to decide on a segmentation. The attention maps were then qualitatively compared to the binary segmentations to investigate if the over-/under-segmentations of the network were on specific areas, therefore highlighting anatomically challenging regions. The attention maps were defined as the activations of the last layer of the nnU-net (i.e. before binarizing).

#### Dosimetric comparison

To assess if the differences between the automatic segmentations and manual segmentations would result in differences in dose-volume parameters, we calculated the D98 and the D90 for both segmentations on the clinical dose distribution used for the treatment. These dose parameters were chosen in accordance with the Embrace II guidelines [1]. The values for the manual segmentations represent the actual treatment parameters for the patients. The dosimetric impact of using automatically segmented structures was defined as the difference between these parameters compared to the clinical values (ΔD90 and ΔD98). The dosimetric impact was also reported as a relative measure by dividing the absolute difference on the dose parameters by the dose parameter on the manual segmentation (ΔD90_rel_ and ΔD98_rel_). The dosimetric results were also compared for patients with different FIGO stage and GTV volume.

### Statistics

The chi-square test for independence was used to confirm that the training, validation and test sets were balanced in terms of FIGO stage. The Kruskal-Wallis H test was used to assess differences among patients of different FIGO stage and GTV volume. If significant differences were found, Dunn’s test with Bonferroni correction was used for the post-hoc analysis. A p-value of 0.05 was considered statistically significant. The SciPy Python package (version 1.5.4) and Python 3.9 were used for the statistical analysis.

## Results

Patients characteristics of our cohort are described in Table 1. No significant differences were found in the distributions of FIGO stage or volume among the training, validation and test sets. The results are shown for 105 out of the 106 cases of the test set. The remaining case corresponded to a patient that had her uterus removed which resulted in a deviating anatomy unseen by the trained network.

**Table 1:** Patients characteristics in the training, validation and test sets

	Training	Validation	Test
**Total**	117	39	39
**Age (years)**			
Mean	53	52	56
Standard deviation	14	15	17
**FIGO stage**			
FIGO I	12 (10.2%)	6 (15.4%)	6 (15.4%)
FIGO II	70 (59.8%)	22 (56.4%)	22 (56.4%)
FIGO III	21 (18.0%)	8 (20.5%)	8 (20.5%)
FIGO IV	9 (7.7%)	3 (7.7%)	3 (7.7%)
Unknown	5 (4.3%)	0	0
**Volume at first BT fraction**			
Less than 2.8 cc	22 (18.8%)	8 (12.8%)	5 ( 20.5%)
between 2.8 and 4.3 cc	7 (6.0%)	7 (17.9%)	7 (17.9%)
between 4.3 and 12.1 cc	53 (42.3%)	12 (30.8%)	13 (33.3%)
More than 12.1 cc	35 (29.9%)	12 (30.8%)	14 (35.9%)
**Histopathological type**			
Squamous cell carcinoma	97 (82.9%)	31 (79.5%)	33 (84.6%)
Adenocarcinoma	16 (13.7%)	6 (15.4%)	4 (10.3%)
Adeno-squamous cell carcinoma	1(0. %)	1 (2.6%)	1 (2.6%)
Non specified/unknown	3 (2.5%)	1 (2.5%)	1 (2.5%)
**External beam radiotherapy scheme**			
23 × 2 Gy	95 (81.2%)	29 (74.4%)	32 (82.0%)
25 × 1.8 Gy	22 (18.8%)	10 (25.6%)	7 (18.0%)

**Fig. 1:**
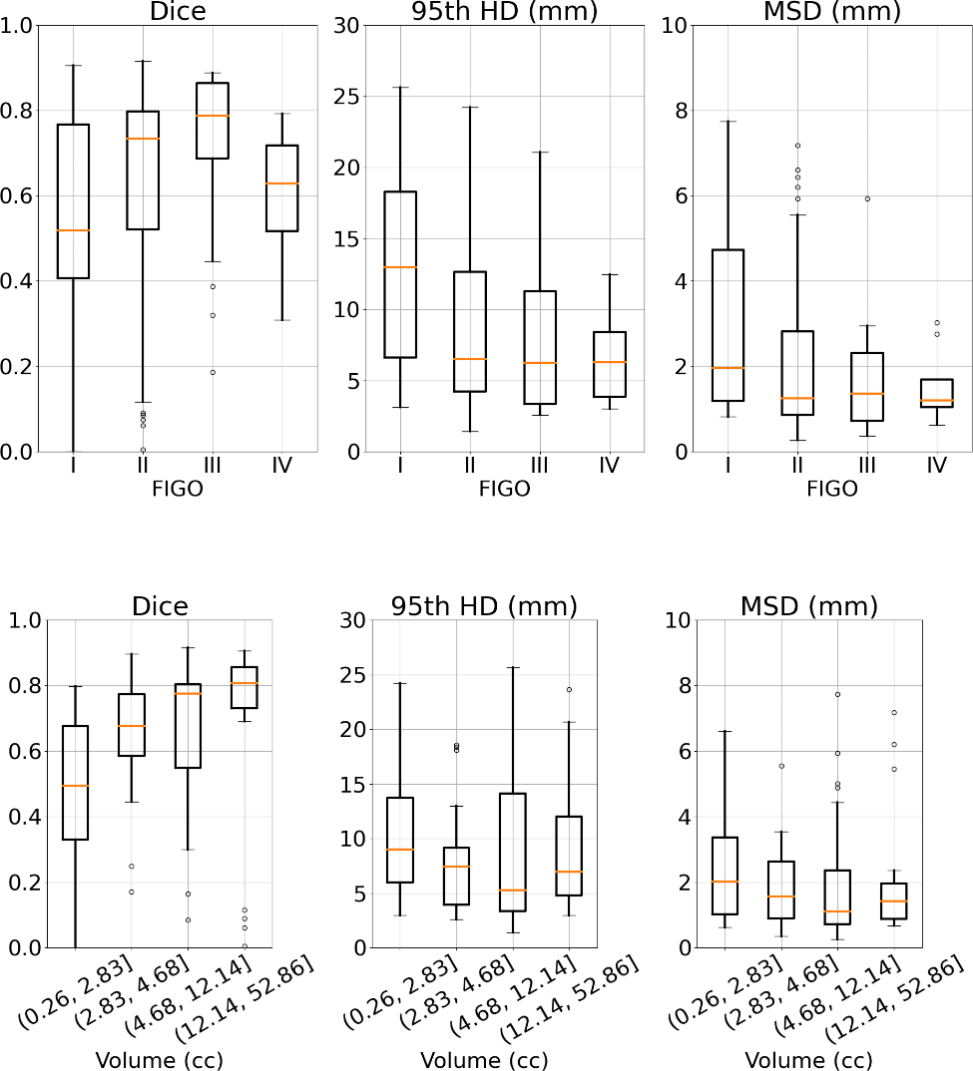
Geometric comparison by FIGO stage (top) and by volume (bottom). Segmentation performance in terms of Dice, 95th HD and MSD and stratified by FIGO stage (I-IV) and volume

The network achieved a median Dice of 0.73 (interquartile range (IQR) = 0.50–0.80), median 95th HD of 6.8 mm (IQR = 4.2 − 12.5 mm) and median MSD of 1.4 mm (IQR = 0.9 − 2.8 mm). When stratifying for FIGO stage (Fig. 1 - top) no significant differences were found among the different subgroups. When comparing for GTV volume (Fig. 1 - bottom) significant differences were found for the case of Dice (p-value < 0.001) but not for the distance-based metrics.

**Fig. 2:**
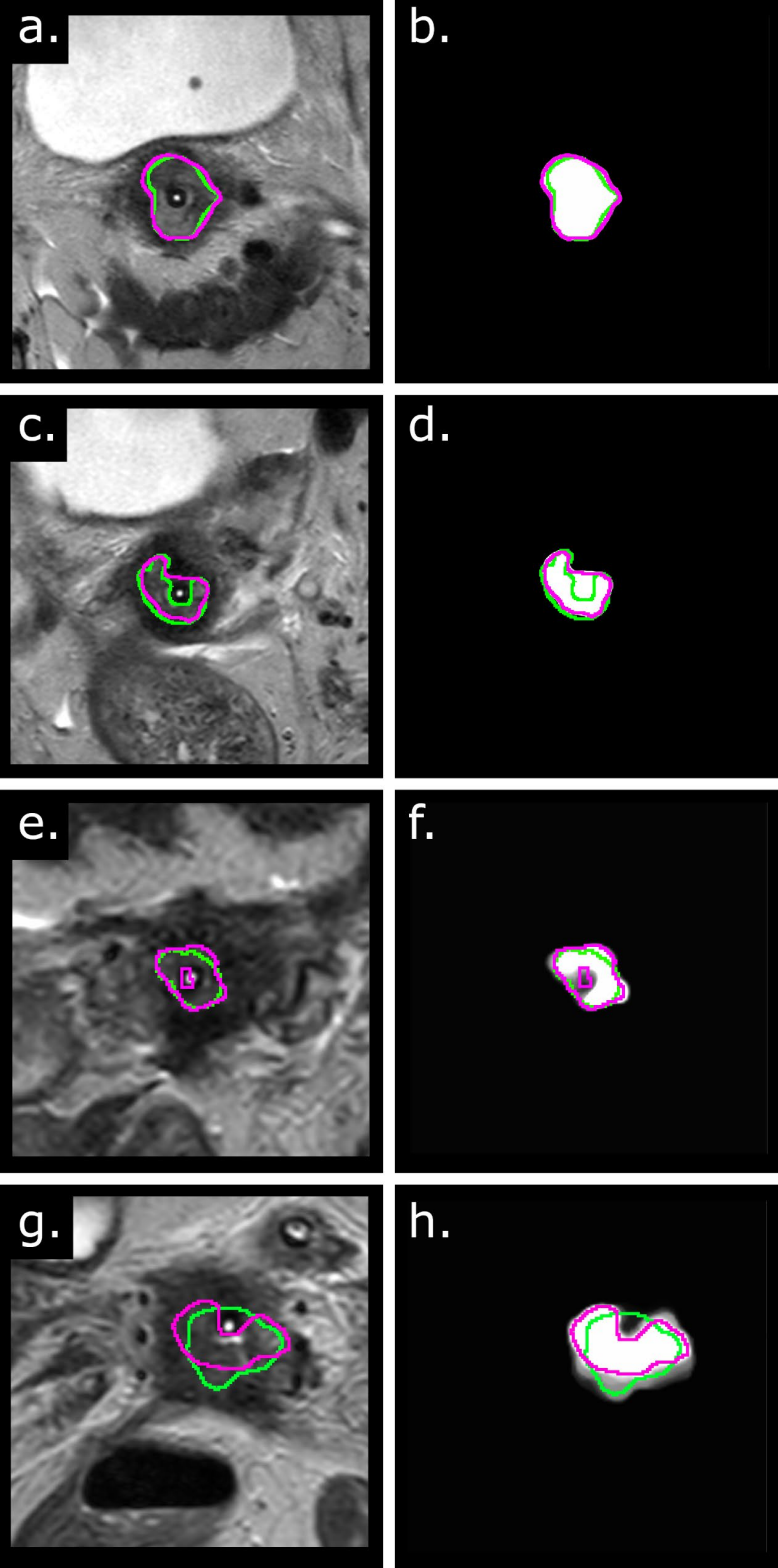
Qualitative results and attention maps. (Left) Examples of the automatic contours (pink) and the manual clinical contour (green) on four different patients. (Right) The corresponding attention maps for the same patients. The examples are sorted by decreasing Dice

Four examples of automatic segmentations are shown in Fig. 2 (a,c,e,g) with the corresponding attention maps (Fig. 2-b,d,f,h). Even though the network under-/over-segmented the GTV for the last three cases (Fig. 2-c,e,g), the error was in the area surrounding the applicator which is an area that is irradiated anyway. In the case 2c, the applicator was segmented by the network but not by the clinician while in the cases 2e and 2f, the clinicians segmented the applicator but the network did not. Furthermore, the attention map highlighted the undersegmented area of the last case (Fig. 2h), meaning that the network looked at that area when deciding the segmentation.

**Fig. 3:**
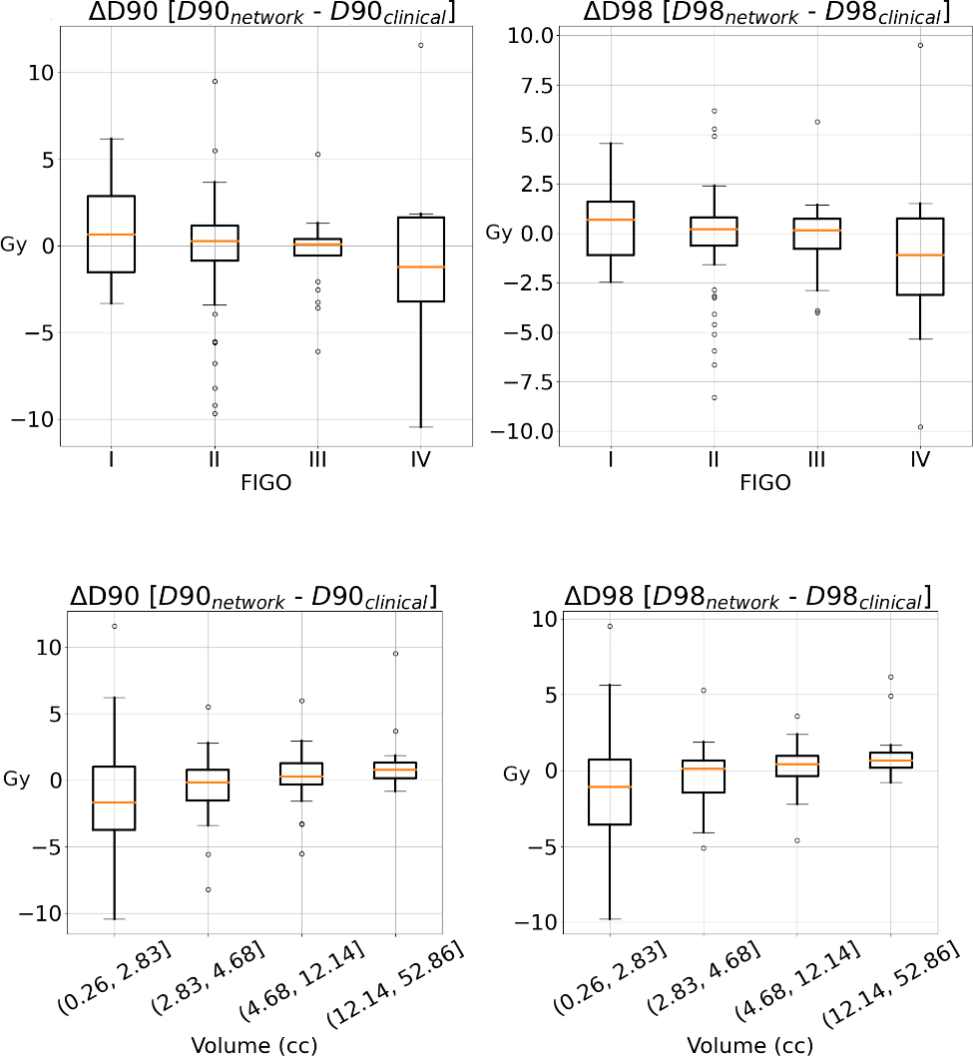
Dosimetric comparison by FIGO stage (top) and by volume (bottom). Dosimetric impact in terms of ΔD90 and ΔD98 stratified by FIGO stage (I-IV) and volume

The median D90 and D98 received by the manually segmented GTV were 12.5 Gy (IQR = 11.1–15.5 Gy) and 10.6 Gy (IQR = 9.4–13.1 Gy), respectively, in line with Embrace guidelines and the GYN GEC-ESTRO recommendations [15]. The resulting ΔD90 and ΔD98 were 0.18 Gy (IQR = -1.38–1.19 Gy) and 0.20 Gy (IQR = -1.10–0.95 Gy), respectively. The median ΔD90_rel_ and ΔD98_rel_ relative differences were 9.6% (QR = 4.2–19.28%) and 8.8% (QR = 0.15–92.5%),respectively. When stratifying for FIGO stage (Fig. 3 - top), no significant differences were observed among the different subgroups per FIGO stage. When comparing the results for GTV volume (Fig. 3 - bottom), a significantly reduced ΔD90 and ΔD98 (p-value < 0.01) was found between the smaller tumors (0.3–2.8 cc) and the largest tumors (12.1–52.9 cc).

## Discussion

In this study we investigated the performance of a state-of-the-art automatic framework to segment the cervical cancer GTV on brachytherapy MR images. We used a cohort of patients that for their treatment were segmented manually by a radiation oncologist and compared these manual segmentations to the automatic segmentations. The comparison was performed geometrically and the impact of differences between automatic and manual delineations on dose-volume parameters of the clinical dose distribution was evaluated.

We achieved improved geometric performance when compared to previously published literature and automatic segmentations yielded a ΔD90 and ΔD98 of less than 0.25 Gy. No significant differences in geometric and dosimetric performance were observed when comparing for FIGO stage. When comparing per volume, decreased performance was observed for smaller tumors both for the Dice coefficient and dosimetrically.

To the best of our knowledge, only Yoganathan et al. [7] studied the automatic segmentation of cervical cancer GTV on brachytherapy images. In their work, they implemented and compared four different CNNs for the segmentation of the targets and organs at risk. The geometric performance of our model was considerably higher than what the authors obtained with the models trained with the axial T2w sequence (Dice: 0.56, 95th HD 9.7 mm). Their models were based on the ResNet and Inception architectures, while we use the nnU-Net, currently the state-of-the-art architecture for medical image segmentation. Additionally, we used a larger cohort.

We observed that the median relative ΔD90_rel_ and ΔD98_rel_ were lower than 10%. Hellebust et al. [16] showed that the relative ΔD90 between different observers was 9.4% for the GTV, meaning that the average difference dosimetric difference between observers is comparable to using the automatic segmentation tool. However, in some of the cases the dosimetric difference was large. These large differences in dosimetric performance can be partially explained by the marked steepness of the brachytherapy dose distributions, which results in that small geometric errors can lead to large differences in dose parameters.

When comparing the results per FIGO stage, no significant differences were found between the different FIGO stages for neither the geometric nor the dosimetric comparisons. A priori, we would have expected the performance to be different between tumors of different FIGO stages because FIGO stage is an important clinical parameter to describe gynecological tumors. One possible reason is that the FIGO stage is defined at the time of diagnosis and consequently does not take into account the regression of the tumor during the external beam radiotherapy treatment, potentially reducing the differences between FIGO stages. On the other hand, when stratifying per GTV volume, significant differences were found for the Dice and for the dosimetric comparisons. For the Dice, the explanation can be rather trivial, because the Dice is defined as the overlap between the two structures and it therefore favors the bigger structures. However, for the dosimetric impact, larger tumors had lower ΔD90 and ΔD98, and less variability, which suggests that smaller tumors may require more accurate automatic segmentation methods than larger tumors.

This work has the following limitations. Firstly, even though our cohort includes a large amount of patients, patients from only one center were included and fewer patients were included for FIGO I and IV. A multi-center validation study is therefore desirable. Secondly, the GTV segmentations used for training and evaluation were manually segmented for clinical practice with the treatment plan in mind, meaning that although the segmentations were clinically acceptable, they may contain geometric errors. These geometric errors could potentially lead the network to reproduce these errors and therefore partially bias our geometric analysis. However, we presented the results in terms of dosimetric impact as well and showed that the dosimetric impact of the automatic segmentations is comparable to that derived from the interobserver variability. Finally, the scope of this work was limited to the GTV automatic segmentation while the HR-CTV and the IR-CTV are also needed for treatment planning. The definition of those structures is intrinsically related to the information of the image before external been radiotherapy (5 weeks before BT) and not only to the information present in the BT image. Therefore in this work we focused solely on the structure that can be found in the BT image.

## Conclusions

In this study we evaluated a state-of-the-art framework for the automatic segmentation of the cervical cancer GTV. The quality of the automatic segmentations improved with respect to previously published works. The automatic segmentations yielded similar dose-volume parameters as the manual segmentations used clinically and differences were comparable to the interobserver variability reported in literature.

## Electronic supplementary material

Below is the link to the electronic supplementary material.

**Supplementary Material 1: Additional File 1.** Description: Extra details on the training procedure. Dictionary information as provided by the nnU-Net in the debug.json.

## Data Availability

The datasets generated and/or analyzed during the current study are not publicly available due to protection of individual patient privacy and the use of an in-house software but are available from the corresponding author on reasonable request.

## List of abbreviations

BT: Brachytherapy
CNN: Convolutional Neural Network
CT: Computed Tomography
Dice: Sørensen–Dice coefficient
FIGO: International Federation of Gynecology and Obstetrics
GTV: Gross Tumor Volume
GYN: Gynaecology
95th HD: 95th Hausdorff Distance
HR-CTV: High Risk Clinical Target Volume
IR-CTV: Intermediate Risk Clinical Target Volume
MSD: Mean surface distance
MRI: Magnetic Resonance Imaging
PET: Positron Emission Tomography
T2w: T2-weighted
