# Supplementary material for: Deep learning for segmentation of the cervical cancer gross tumor volume on magnetic resonance imaging for brachytherapy
Source: Radiat Oncol. 2023 May 29;18:91. doi: 10.1186/s13014-023-02283-8 (PMC10227985; doi:10.1186/s13014-023-02283-8)
Supplement: Supplementary file 1 — Supplementary Material 1: Additional File 1. Description: Extra details on the training procedure. Dictionary information as provided by the nnU-Net in the debug.json. [file 13014_2023_2283_MOESM1_ESM.docx]

**Additional File 1. Extra details on the training procedure.**

Dictionary information as provided by the nnU-Net in the debug.json. The specific paths are redacted.

{

"all_tr_losses": "[]",

"all_val_eval_metrics": "[]",

"all_val_losses": "[]",

"all_val_losses_tr_mode": "[]",

"also_val_in_tr_mode": "False",

"amp_grad_scaler": "None",

"base_num_features": "32",

"basic_generator_patch_size": "[ 24 376 376]",

"batch_dice": "True",

"batch_size": "2",

"best_MA_tr_loss_for_patience": "None",

"best_epoch_based_on_MA_tr_loss": "None",

"best_val_eval_criterion_MA": "None",

"classes": "[1]",

"conv_per_stage": "2",

"data_aug_params": "{'selected_data_channels': None, 'selected_seg_channels': [0], 'do_elastic': False, 'elastic_deform_alpha': (0.0, 200.0), 'elastic_deform_sigma': (9.0, 13.0), 'p_eldef': 0.2, 'do_scaling': True, 'scale_range': (0.7, 1.4), 'independent_scale_factor_for_each_axis': False, 'p_independent_scale_per_axis': 1, 'p_scale': 0.2, 'do_rotation': True, 'rotation_x': (-3.141592653589793, 3.141592653589793), 'rotation_y': (-0.5235987755982988, 0.5235987755982988), 'rotation_z': (-0.5235987755982988, 0.5235987755982988), 'rotation_p_per_axis': 1, 'p_rot': 0.2, 'random_crop': False, 'random_crop_dist_to_border': None, 'do_gamma': True, 'gamma_retain_stats': True, 'gamma_range': (0.7, 1.5), 'p_gamma': 0.3, 'do_mirror': True, 'mirror_axes': (0, 1, 2), 'dummy_2D': True, 'mask_was_used_for_normalization': OrderedDict([(0, True)]), 'border_mode_data': 'constant', 'all_segmentation_labels': None, 'move_last_seg_chanel_to_data': False, 'cascade_do_cascade_augmentations': False, 'cascade_random_binary_transform_p': 0.4, 'cascade_random_binary_transform_p_per_label': 1, 'cascade_random_binary_transform_size': (1, 8), 'cascade_remove_conn_comp_p': 0.2, 'cascade_remove_conn_comp_max_size_percent_threshold': 0.15, 'cascade_remove_conn_comp_fill_with_other_class_p': 0.0, 'do_additive_brightness': False, 'additive_brightness_p_per_sample': 0.15, 'additive_brightness_p_per_channel': 0.5, 'additive_brightness_mu': 0.0, 'additive_brightness_sigma': 0.1, 'num_threads': 12, 'num_cached_per_thread': 2, 'patch_size_for_spatialtransform': array([ 24, 224, 320])}",

"dataset_directory": "/project/outeiral/data_seg/nnUNet_preprocessed/Task517_Cervix",

"deep_supervision_scales": "[[1, 1, 1], [1.0, 0.5, 0.5], [1.0, 0.25, 0.25], [0.5, 0.125, 0.125], [0.25, 0.0625, 0.0625], [0.25, 0.03125, 0.03125]]",

"deterministic": "False",

"dl_tr": "<nnunet.training.dataloading.dataset_loading.DataLoader3D object at 0x14d57c92bee0>",

"dl_val": "<nnunet.training.dataloading.dataset_loading.DataLoader3D object at 0x14d57c92bf10>",

"do_dummy_2D_aug": "True",

"ds_loss_weights": "[0.51612903 0.25806452 0.12903226 0.06451613 0.03225806 0. ]",

"epoch": "0",

"experiment_name": "nnUNetTrainerV2",

"fold": "0",

"folder_with_preprocessed_data": "/X/nnUNet_preprocessed/Task517_Cervix/nnUNetData_plans_v2.1_stage1",

"fp16": "True",

"gt_niftis_folder": "/X/nnUNet_preprocessed/Task517_Cervix/gt_segmentations",

"inference_pad_border_mode": "constant",

"inference_pad_kwargs": "{'constant_values': 0}",

"init_args": "('/X/nnUNet_preprocessed/Task517_Cervix/nnUNetPlansv2.1_plans_3D.pkl', 0, '/X/nnUNet_trained_models/nnUNet/3d_fullres/Task517_Cervix/nnUNetTrainerV2__nnUNetPlansv2.1', '/X/nnUNet_preprocessed/Task517_Cervix', True, 1, True, False, True)",

"initial_lr": "0.01",

"log_file": "/X/nnUNet_trained_models/nnUNet/3d_fullres/Task517_Cervix/nnUNetTrainerV2__nnUNetPlansv2.1/fold_0/training_log_2022_9_9_17_34_13.txt",

"lr_scheduler": "None",

"lr_scheduler_eps": "0.001",

"lr_scheduler_patience": "30",

"lr_threshold": "1e-06",

"max_num_epochs": "1000",

"min_region_size_per_class": "None",

"min_size_per_class": "None",

"net_conv_kernel_sizes": "[[1, 3, 3], [1, 3, 3], [3, 3, 3], [3, 3, 3], [3, 3, 3], [3, 3, 3], [3, 3, 3]]",

"net_num_pool_op_kernel_sizes": "[[1, 2, 2], [1, 2, 2], [2, 2, 2], [2, 2, 2], [1, 2, 2], [1, 1, 2]]",

"net_pool_per_axis": "[2, 5, 6]",

"normalization_schemes": "OrderedDict([(0, 'nonCT')])",

"num_batches_per_epoch": "250",

"num_classes": "2",

"num_input_channels": "1",

"num_val_batches_per_epoch": "50",

"online_eval_fn": "[]",

"online_eval_foreground_dc": "[]",

"online_eval_fp": "[]",

"online_eval_tp": "[]",

"only_keep_largest_connected_component": "None",

"optimizer": "SGD (\nParameter Group 0\n dampening: 0\n lr: 0.01\n momentum: 0.99\n nesterov: True\n weight_decay: 3e-05\n)",

"output_folder": "/X/nnUNet_trained_models/nnUNet/3d_fullres/Task517_Cervix/nnUNetTrainerV2__nnUNetPlansv2.1/fold_0",

"output_folder_base": /X/nnUNet_trained_models/nnUNet/3d_fullres/Task517_Cervix/nnUNetTrainerV2__nnUNetPlansv2.1",

"oversample_foreground_percent": "0.33",

"pad_all_sides": "None",

"patch_size": "[ 24 224 320]",

"patience": "50",

"pin_memory": "True",

"plans_file": /X/nnUNet_preprocessed/Task517_Cervix/nnUNetPlansv2.1_plans_3D.pkl",

"regions_class_order": "None",

"save_best_checkpoint": "True",

"save_every": "50",

"save_final_checkpoint": "True",

"save_intermediate_checkpoints": "True",

"save_latest_only": "True",

"stage": "1",

"threeD": "True",

"tr_gen": "<batchgenerators.dataloading.multi_threaded_augmenter.MultiThreadedAugmenter object at 0x14d57c8dd8e0>",

"train_loss_MA": "None",

"train_loss_MA_alpha": "0.93",

"train_loss_MA_eps": "0.0005",

"transpose_backward": "[0, 1, 2]",

"transpose_forward": "[0, 1, 2]",

"unpack_data": "True",

"use_mask_for_norm": "OrderedDict([(0, True)])",

"use_progress_bar": "False",

"val_eval_criterion_MA": "None",

"val_eval_criterion_alpha": "0.9",

"val_gen": "<batchgenerators.dataloading.multi_threaded_augmenter.MultiThreadedAugmenter object at 0x14d57c8dddc0>",

"was_initialized": "True",

"weight_decay": "3e-05"

}
